# A Spatial Data Infrastructure for Environmental Noise Data in Europe †

**DOI:** 10.3390/ijerph14070726

**Published:** 2017-07-06

**Authors:** Andrej Abramic, Alexander Kotsev, Vlado Cetl, Stylianos Kephalopoulos, Marco Paviotti

**Affiliations:** 1 ECOAQUA University Institute, Scientific and Technological Marine Park, University of Las Palmas de Gran Canaria, 35001 Las Palmas, Spain; andrej.abramic@ulpgc.es; 2 Directorate General “Joint Research Centre”, European Commission, 21027 Ispra, Italy; alexander.kotsev@ec.europa.eu (A.K.); stylianos.kephalopoulos@ec.europa.eu (S.K.); 3 Directorate General “Environment”, European Commission, 1049 Brussels, Belgium; marco.paviotti@ec.europa.eu

**Keywords:** noise data, Environmental Noise Directive, INSPIRE, spatial data infrastructure, e-reporting

## Abstract

Access to high quality data is essential in order to better understand the environmental and health impact of noise in an increasingly urbanised world. This paper analyses how recent developments of spatial data infrastructures in Europe can significantly improve the utilization of data and streamline reporting on a pan-European scale. The Infrastructure for Spatial Information in the European Community (INSPIRE), and Environmental Noise Directive (END) described in this manuscript provide principles for data management that, once applied, would lead to a better understanding of the state of environmental noise. Furthermore, shared, harmonised and easily discoverable environmental spatial data, required by the INSPIRE, would also support the data collection needed for the assessment and development of strategic noise maps. Action plans designed by the EU Member States to reduce noise and mitigate related effects can be shared to the public through already established nodes of the European spatial data infrastructure. Finally, data flows regarding reporting on the state of environment and END implementation to the European level can benefit by applying a decentralised e-reporting service oriented infrastructure. This would allow reported data to be maintained, frequently updated and enable pooling of information from/to other relevant and interrelated domains such as air quality, transportation, human health, population, marine environment or biodiversity. We describe those processes and provide a use case in which noise data from two neighbouring European countries are mapped to common data specifications, defined by INSPIRE, thus ensuring interoperability and harmonisation.

## 1. Introduction

Environmental noise is increasingly recognized globally as an issue of concern. At the same time substantial volumes of high quality data are required in order to (i) properly estimate the population exposure levels; (ii) assess the impact on human health and the environment; and (iii) compare across different regions and countries in an increasingly urbanized world. The availability and usability of such data on a national level is challenging, but if extended to the international level it becomes even harder. There are numerous difficulties associated with the exchange and joint use of noise data on an international scale. They are related, among others, to the different algorithms and modelling decisions, heterogeneous data structures, and multiple languages [1].

Noise is an issue of major concern for globally (especially in urban areas it affects a large number of people) and noise reduction has increasingly become a focus for legislation and management. Increasingly there is evidence of the adverse effect of noise and it’s relation to cardiovascular diseases [2,3]. The burden of disease from environmental noise in Europe was recently estimated by WHO-JRC [4] accounts for 1.6 million healthy life years lost every year in urban areas in Western Europe. According to preliminary results from the project ‘Environmental Burden of Disease in Europe’, run in six European countries [5], and reported at the WHO Ministerial Conference 2010, traffic noise was ranked second among the selected environmental stressors evaluated in terms of their public health impact.

Further, the trend is that noise exposure is increasing in Europe compared to other stressors (e.g., exposures to second hand smoke, dioxins and benzene), which are declining.

Noise affects people in both physiological and psychological ways, interfering with basic activities such as sleep, rest, work, study and communication. Epidemiological evidence indicates that those chronically exposed to high levels of environmental noise have an increased risk of cardiovascular diseases such as myocardial infarction as supported by the estimated 10–20.000 premature deaths due to environmental noise every year in the EU-27. Thus, noise pollution affects the human health and well-being with increasing expenditures due to medical treatment and reduced productivity at work.

In the European Union (EU), with its Member States (MS), a reliable estimation of the exposure to environmental noise is a pre-requisite to support and evaluate an informed policy on noise reduction. European legislation is put in place—European Directive on the Assessment and Management of Environmental Noise (2002/49/EC) (END) [6]—with the overall objective to establish a common approach to assess the exposure to environmental noise. A comprehensive overview of both the calculation and mapping methods within the Directive are provided by Murphy and King [7]. Within this context, in order to obtain consistent and comparable figures on the number of people being exposed to noise levels within and across EU MS, the European Commission (EC) has developed a common methodological framework for the assessment of environmental noise and its impact on human health (CNOSSOS-EU) [8]. CNOSSOS-EU aims to enable a consistent and accurate reporting of strategic noise maps (including exposure of populations) by MS in accordance to their obligations under the END starting from 2018 onwards. The European Environmental Agency (EEA) has put in place a system with the aim of monitoring the state of environmental noise throughout the European Union [9].

In accordance with the above-mentioned legislation, countries are required to produce strategic noise maps (data) on a five-year basis for all major roads, railways, airports and agglomerations. The outcome of these maps is then used by the competent authorities to identify priorities for action planning (aimed at reducing or avoiding exposure to harmful noise levels), and by the European Commission (EC) to assess exposure to noise, elaborate pan-European policy, and inform the general public. The objective of our paper is to propose an approach which can enhance the usability and access to environmental noise data through the application of a coherent reporting mechanism. In our proposal noise data is seen within the broader context of an environmental spatial data infrastructure (SDI). We consider that SDIs provide a unique opportunity for combining data across thematic domains [1,10] and countries [11]. The overarching context of this work is defined by the largest single regional SDI in the world, being established through the implementation of the INSPIRE Directive (Infrastructure for Spatial Information in the European Community, 2007/2/EC) [12]. This manuscript therefore elaborates on the potential for benefiting from (i) the harmonisation efforts within the CNOSSOS-EU process in Europe by (ii) using INSPIRE for unlocking, utilisation and reuse of relevant spatial data. There are opportunities and synergies that go beyond environmental noise. Considering the complexity and heterogeneity of the situation in Europe with regards to noise data, within the broader context of Digital Earth [13], the activities described here should be seen as a valuable example for the rest of the world.

Structurally the manuscript is organized into four sections. The first defines the context and theoretical foundations, and in particular the concept of a SDI and the current situation with respect to environmental noise data in Europe. Section 2 goes into further detail and illustrates the value added from data harmonisation based on INSPIRE. We continue with a use-case on transforming data from two European Member States (Section 3). The article closes with our main conclusions and an outline of our future work.

### 1.1. Environmental Noise Data in Europe

Approximately 22 percent of the population of the EU is exposed to traffic-related noise exceeding 55 decibels [14]. That is why the END [6] defines a common approach intended to avoid, prevent or reduce on a prioritised basis the harmful effects, including annoyance, due to the exposure to environmental noise. The EC assessed the degree of comparability of the results generated through different methods after an initial round of strategic noise mapping (2006–2007) and established that, in many cases, the assessment methods used by MS differ significantly from the interim methods [8]. Other assessments have shown that differences in methodological approaches made it difficult (if not impossible) to obtain consistent and comparable figures on the number of people being exposed to noise levels within and across EU MS ([15,16]). Difficulties relate, inter alia, to: (i) incompleteness of the reporting of strategic noise maps by MS; (ii) the different quality and format of data reported at EU level; (iii) the different assessment methods used; (iv) the different strategies adopted concerning the selection of e.g., roads to be mapped; (v) the distribution of the populations and dwellings within buildings and (vi) the unavailability or reliable dose—response curves required for health impact assessment.

Lately, a report on the implementation of END in accordance with Article 11 of Directive 2002/49/EC, released in 2011 (COM(2011) 321 final) [17] defines four main directions in which the implementation of the Directive can be improved.

Firstly, EC refers to the need to finalising the harmonised framework for assessing noise exposure in Europe that started in the 2008 by the CNOSSOS-EU project led by the Joint Research Centre [1,7]. In 2014 the Annex II of the END was revised on the basis of the CNOSSOS-EU methods and is under scrutiny by the European Parliament before it becomes new regulation in the EU member states starting from 2018.

Secondly, the Directive provides an implementing framework for reducing the environmental noise impact, and therefore main issues could be approached by different methods. From a legal standpoint it left some space for different interpretation of the provisions included in the legal text and allowed for the possibility to use as interim solution different approaches for noise assessment (till CNOSSOS-EU will be legally enforced) and other implementation aspects.

Thirdly, implementation of the legislation on environmental noise deals with a number of cross cutting issues such as the needs for coordination and integration of air quality and noise management that would imply common data collection, coordinated assessments, action plans, reporting to the EC/EEA and dissemination of information to public. A similar activity has already been done in the domain of air quality for e-reporting of EU MS according to the requirements of the Ambient Air Quality Directive (2008/50/EC). The undertaken approach is documented by [18] and the lessons learned are provided in [19].

The fourth issue identified is related to the reporting cycles of the END. The reporting obligations, in some cases, create an administrative burden without added value that possibly could be decreased by the development of an appropriate e-reporting system.

The EC carried out a public online consultation to collect the views of citizens, non-governmental organisations and other interested stakeholders, addressing specifically the relevance, effectiveness, efficiency and EU-added-value of the END. The consultation [20] was open from December 2015 till March 2016 and the overall findings show that noise remains a relevant issue. The perception of the majority of respondents is that the END was useful from a legislative point of view, but at the same time citizens were in most cases not consulted on action plans. In addition, policy interventions on national level were inefficient. Many respondents commented that action plans were done only ‘pro-forma’ and no actual measures were taken. The consultation confirmed that there is definitely a consensus to keep, and even strengthen, the EU legislation on environmental noise, with the setting of EU noise limits, or to a slightly lesser extent EU noise targets, being a missing element of the Directive. That is why, from our perspective the EU policy including END should be stronger and more ambitious.

### 1.2. Shared Environmental Information System

Effective policy making concerning environmental noise largely depends on the availability and quality of underpinning data and the systems needed to share this information between all actors (i.e., policy makers, health authorities, consultants, and other relevant stakeholders). Despite the long tradition of environmental data sharing in Europe there are still numerous challenges not permitting an easy use of environmental information across borders and loosely coupled thematic domains. This is caused by numerous interdependent factors, inherent to one of the key characteristics of Europe—its diversity. The factors contributing to this diversity include: the 24 official languages used in EU, the completely different workflows between countries, the broad diversity of proprietary and open source tools in use, differences in the culture and traditions of sharing data, etc. In response to that the European Commission together with EU Member States is working on the establishment of a Shared Environmental Information System—SEIS [21] and laying down the foundation of an Infrastructure for Spatial Information in Europe—INSPIRE [12]. The issues addressed by SEIS and INSPIRE are common to many countries and domains, and are in accordance with [19] typically a result of:

- *Data heterogeneity.* Infrastructures for the collection, maintenance and exchange of information in Europe are very different from each other. The political decision for the establishment of INSPIRE through a legislative approach provides the means for facing the challenge of setting-up and using a common infrastructure out of a transparent and inclusive approach which takes on board the views of all European member state stakeholders and institutions.
- *Situations of lock-in.* Despite the long lasting efforts for unlocking public data, considerable portions are still only available in data ‘silos’, which limit their use and reuse.
- *Lack of trust.* Both SEIS and INSPIRE aim to change the traditional model of data storage and maintenance and to provide the means for multiple use of same datasets. Making that possible would strongly depend apart from compliance with legally binding obligations also on mutual trust and willingness to share environmental data.
- *Limited use of standards.* Adoption of commonly agreed standards for encoding and transmission of information between institutions in Europe is still limited. At the same time the use of standards would provide a transparent approach for data governance and interoperability. That is why international standards are the ‘backbone’ of INSPIRE legal framework.

### 1.3. Infrastructure for Spatial Information in Europe

**Figure 1:**
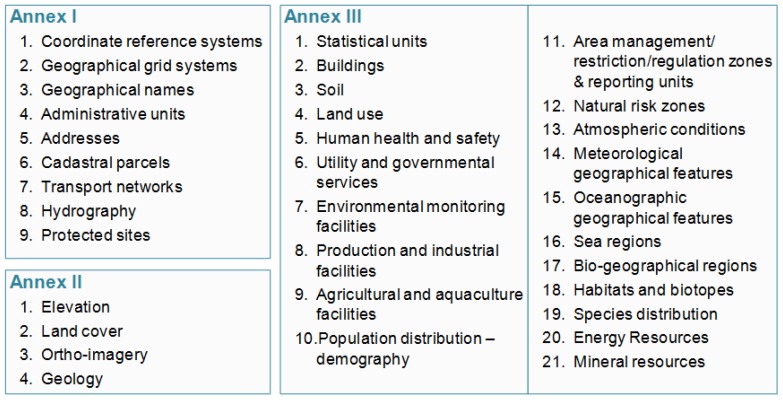
The Infrastructure for Spatial Information in the European Community (INSPIRE) legal acts and data specifications harmonise spatial objects, key attributes and exchange formats of 34 data themes.

The INSPIRE Directive [12], within the broader context of SEIS, aims to provide harmonised, high quality spatial information available to support environmental policies along with policies or activities which may have an implicit impact on the environment in Europe. This legally mandated initiative paves the ground for European countries to build a common Spatial Data Infrastructure (SDI). INSPIRE is organised through 34 data themes (see Figure 1) and a set of standardised web-based services aiming to significantly improve the access and usage of data. Developed through a transparent and participatory process, INSPIRE will enable the sharing of environmental spatial information among public sector organisations and better facilitate public access to spatial information across Europe. The implementation process of the Directive should be completed by the end of 2021 and adopts a step-wise approach where the overall process is improved by feeding the discoveries of implementers into existing technical guidance documentation. Legislatively INSPIRE is divided into the following components: metadata, network services, interoperability of spatial data sets and services, data sharing, monitoring and reporting. It is worth noting that INSPIRE uses international standards as building blocks of the European interoperability infrastructure, thus by implementing it national authorities add value to their existing systems through harmonising them. Significant share of the standards which are being used in INSPIRE are created within the Open Geospatial Consortium (OGC) and the International Standardization Organization (ISO).

In October 2013, the European Commission adopted amendment for interoperability of spatial data sets and services for Annex II and III data themes what is probably the world’s single largest data harmonisation effort related to environmental information. Hundreds, if not thousands, of experts from across Europe are involved in this process. They have been working together for over a decade to agree on common definitions in a cross-cutting setting, focusing on important policy areas such as air quality, energy, climate change, biodiversity, human health, etc. (Figure 1). This piece of legislation complements other INSPIRE legal acts and standards. Together they form the basis of what the INSPIRE Directive envisions. Now that most documents needed for the establishment of the infrastructure are agreed, the implementation continues with the main responsibility on the Member States side.

Both SEIS and INSPIRE are organized around several interdependent principles [21], that if followed, would considerably improve the situation with regards to the access and usability of spatial data on noise. The principles include:

- Data should be collected only once and kept where it can be maintained most effectively.
- It should be possible to combine seamless spatial information from different sources across Europe and share it with many users and applications.
- It should be possible for information collected at one level/scale to be shared with all levels/scales; detailed for thorough investigations, general for strategic purposes.
- Spatial information needed for good governance at all levels should be readily and transparently available, fully available to the general public, to also enable citizen participation.
- Easy to find what Spatial information is available, how it can be used to meet a particular need, and under which conditions it can be acquired and used.
- Spatial data should become easy to understand and interpret because it can be visualized within the appropriate context selected in a user-friendly way
- Supported through common, free and open software standards.

## 2. A Spatial Data Infrastructures Perspective for Environmental Noise Data

**Figure 2:**
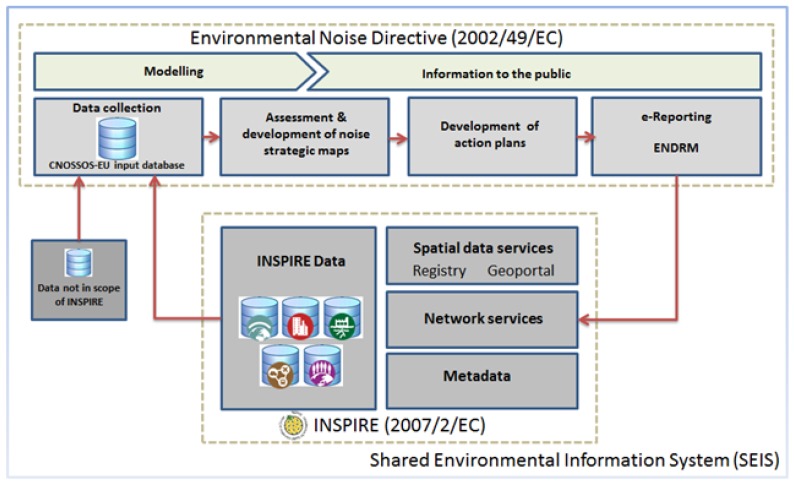
Interdependence between Environmental Noise Directive (END) and INSPIRE.

The implementation of INSPIRE as a European SDI framework, following the principles mentioned above, would contribute to the exchange and comparability of environmental noise data in Europe [22]. INSPIRE already provides building blocks (Figure 2) which can be reused in the context of noise to encode data in a similar manner, thus achieving semantic interoperability. Moreover, web services following common specifications should expose data on the web.

The next subsections provide an overview of the benefit from adopting INSPIRE.

### 2.1. INSPIRE and Noise Modelling

**Figure 3:**
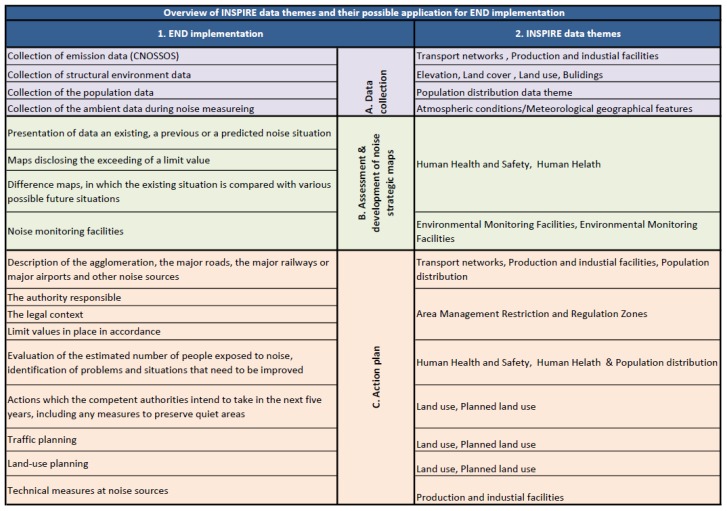
Theoretical mapping between INSPIRE data themes and END Implementation.

Spatial information provided through INSPIRE can support the CNOSSOS-EU common modelling and the establishment of a joint international assessment methodological framework. This would be done, as both CNOSSOS-EU and INSPIRE have the potential to provide a harmonised and coherent approach towards the assessment of noise levels from major noise sources such as road traffic, railway traffic, aircraft and industrial premises. Statistical data on traffic is not within the scope of INSPIRE, but the resultant SDI would nonetheless provide significant amounts of information, thematically related to the identification, monitoring and assessment of harmful noise effects that could potentially become a part of the CNOSSOS–EU database (Figure 3).

### 2.2. Support for Data Exchange and Reporting

The END provides a framework for assessing and reducing the noise impact. The harmonisation of strategic mapping and development of appropriate action plans required for the mitigation of harmful noise effects is equally important to the harmonisation of assessment methods. That is why spatial information regarding the agglomerations, evaluation of population exposed to noise, action plans and related planning, required by the END themes listed in Figure 3 should be harmonised through adoption of the already established INSPIRE data models, making it easier to integrate, and if necessary to compare interoperable data from different sources.

**Figure 4:**
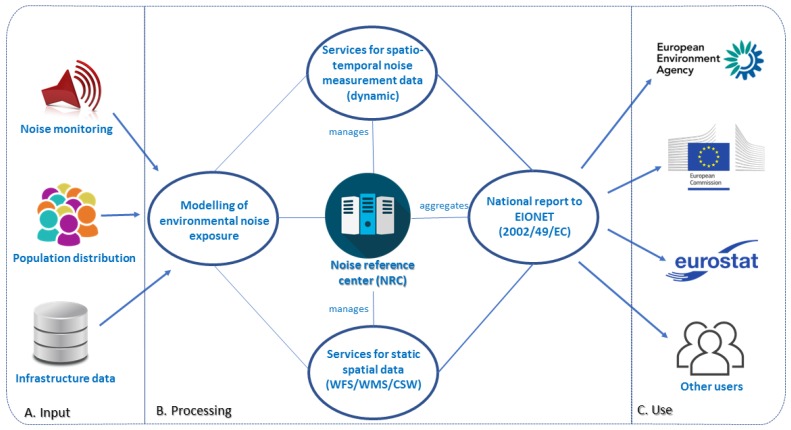
A scheme for potential noise data flows after aligning to INSPIRE compliant e-reporting requirements, as applied in the air quality domain. Modified from: [19].

Furthermore, network services for discovering (metadata), viewing and downloading of spatial data [23] are established in INSPIRE. Those services, developed within national SDIs, in accordance with SEIS, so that data and information are located where they are best managed and maintained. Services should provide the foundations for e-reporting that would enable data flows to European institutions (Figure 4) and constantly updated data and required assessment. Within this context, it is increasingly recognized from the experience the EU MS acquired during the first round of noise mapping that the current data exchange practices and the e-reporting system put in place by EEA would be improved through INSPIRE. Early in 2012, the EEA published specific guidance for delivering environmental noise data using the END Reporting Mechanism (ENDRM) [24]. The EEA guidance notes that relevant elements of ENDRM have been formatted to meet the requirements of INSPIRE. This includes the use of the ETRS89 positional referencing system [25] and the use of spatial metadata standards to accommodate delivery of noise maps, source locations, agglomeration boundaries and action planning areas, including zones delimited as quiet areas. The specified metadata standards for spatial data are those currently adopted by the EEA and proposed for future use within INSPIRE. They are based around a profile of ISO19115. EEA states that its standards will be regularly updated and the standards set by the INSPIRE directive will be followed.

It also happened but rarely to already employ the INSPIRE standard when automating the noise mapping process. One of the most advanced approaches is the one taken by the state of Nordrhein-Westfalen in Germany, which uses an integrated database-GIS-calculation-web solution [9,26,27].

### 2.3. Cross-Domain Interoperability

The need to improve synergies between noise management and other EU policies, for example air quality, transport and INSPIRE (collection of spatial information) is among the recommendations issued by the European Parliament’s report “Towards a Comprehensive Noise Strategy” [28].

INSPIRE compliant e-reporting on air quality in Europe is in a stage which is more advanced with Member State public sector authorities, having to align existing dataflow and encode and report information to the EEA in a way compliant to both INSPIRE and the Air Quality Directive [18,19]. This provides a unique opportunity for aligning noise with air quality data on a European level, as to improve the implementing process and avoid facing similar and already previously identified issues.

Geographic Information Systems (GIS) are the de facto standard used by noise modellers for the preparation of quantifiable data and noise maps that can further be used within action plans. Some of the typical operations include (i) data collection and preparation (i.e., spatial input data needed to run the noise model software); (ii) analysis of noise level distributions and (iii) dissemination of information to public (i.e., main results of noise mapping and action plans). GIS doubtlessly offer a powerful toolbox, however they do not solve the issues associated with data interoperability. The ways in which spatial and attributive data are structured varies considerably from one package to another. A clear case that should be investigated on a case by case basis is the semantics used in the resultant databases, as well as the input data. Within this context, the suitability of heterogeneous types of input data for noise modelling purposes must be evaluated, considering among other issues the possibilities for data exchange [16]. There are many examples of attributes difficult to combine because they are codified in very different ways. For example building heights might be encoded differently depending on the application domain (e.g., expressed in metres, floors, high/middle/low, etc.). From that point of view INSPIRE provides an excellent opportunity as it’s implementation would put order in how data are encoded.

## 3. Data Harmonisation Use-Case

In the following section we provide a use case, where we try to combine noise data which is already been reported to the EEA by EU Member States into a GIS. We used test data from Germany (Saxony) and Poland, as both countries have reported in 2013 data for END to the EEA. The source of the data is available at the EEA data repository [29].

**Figure 5:**
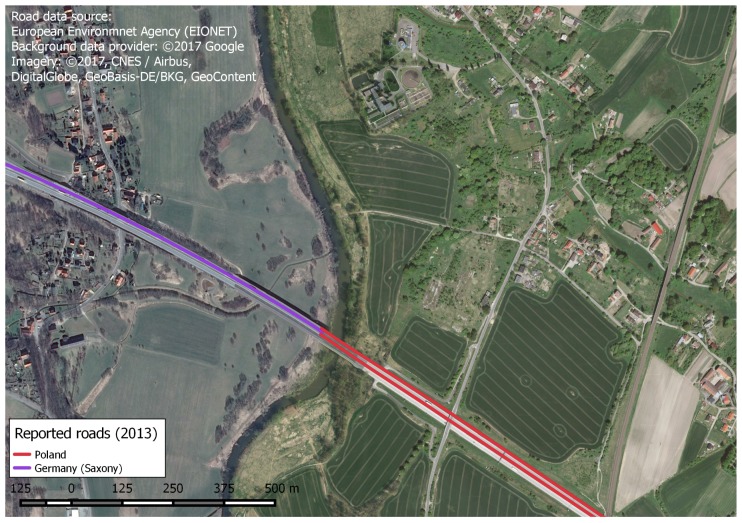
Mismatch of road network geometry reported to the EEA by Germany and Poland in 2013.

Taking the road network (vector line features), as input we found considerable differences in the way it was provided by the two countries in terms of geometry and attributes. Germany reported the road network as single lines, while in Poland data is represented in a network model with multiple lines for different traffic directions of motorways (Figure 5). This, combined with the heterogeneous attributive information leads to the question on the usability of data as-is. From our perspective it would be hard, if at all possible, to jointly use this data without considerable transformation.

**Figure 6:**
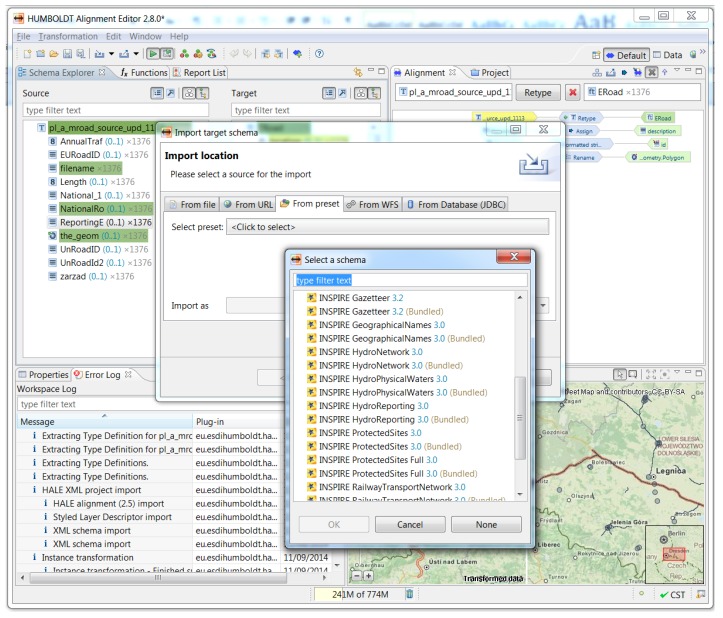
Mapping between source and target (INSPIRE compliant) schema in HUMBOLDT Alignment Editor (HALE).

To prove the added value of INSPIRE, particularly for harmonising data across borders for the purposes of comparable assessment of environmental noise, we transformed the data for both Saxony and Poland through the use of an open source extract-transform-load (ETL) tool called HUMBOLDT Alignment Editor (HALE). The product is described by Reitz and Templar [30] and provides functionality for mapping between source and target application schemas. Following the mapping it is possible to transform data in accordance with the INSPIRE models, as defined in Section 1.3 of this paper. Within HALE we imported both data sources, and mapped them (Figure 6) according to the INSPIRE common data specifications. The output data files can afterwards be combined and used together. Extending to a European level would result in harmonised noise data, which would be a solid basis for a better understanding of the problem, as well as for informed decision making and improved policy making.

## 4. Conclusions

During the first END reporting cycle, data were collected that allowed the assessment of individual exposure to environmental noise from four major sources (road traffic, railway traffic, aircraft traffic and industrial premises). Action plans were developed by the EU Member States and all were communicated to the European Commission through various data flows, defined by the EEA. Still, the reported information was difficult to compare or combine, as it was produced without using common specific rules and established methodologies. Development of the common noise assessment methodological framework in Europe (CNOSSOS-EU project, [1]) represents one concrete step forward in the implementation of the END which will help to overcome existing inconsistent approaches in the strategic noise mapping in Europe and allow for better noise data comparability across Europe. Additionally, consistent spatial information, supported by implementation of the SEIS principles and development of the European SDI, is expected to considerably support the implementation of the END requirements and a sustainable noise data management. Ensuring that (i) data are properly documented through metadata; (ii) harmonised and (iii) served through INSPIRE services would add to the benefits from using the CNOSSOS-EU harmonization framework and the EEA’s reporting mechanism (ENDRM), improve synergies with Ambient Air Quality Directive’s implementation and finally provide a roadmap for a decentralized reporting system. One of the important use cases that led to the creation of INSPIRE 10 years ago was to facilitate electronic reporting (e-reporting). That is why many of the themes listed in the Annexes of the INSPIRE Directive refer to relevant geospatial data which stem from EU environment legislation (e.g., hydrography in Annex I refers to the Water Framework Directive). Furthermore, during the development of the Implementing Rules for data interoperability, the existing reporting legislation and flows were initially analysed and used as drivers for the development of the harmonised data models. INSPIRE is a huge technical and organisational effort linking together the national and sub-national data infrastructures of 28 EU countries, in 24 languages, and covering 34 broad data themes. Several data themes directly support END reporting: Area management/restriction/regulation zones and reporting units, Atmospheric conditions, Buildings, Elevation, Environmental monitoring Facilities, Land cover, Land use, Population distribution, Production and industrial facilities, Transport networks, Utility and governmental services. After having (i) clarified the European context in Section 1 and Section 2; and (ii) successfully implemented a data transformation procedure, described in Section 3 of this manuscript, we envisage the following way ahead for a streamlined noise data infrastructure based on INSPIRE:

- Establishment of an INSPIRE/END e-reporting pilot project with the involvement of data providers and relevant pan-European actors
- Identification of possible extensions of existing INSPIRE data models in order to accommodate data needed for END purposes
- Use of interoperable network services in a Service Oriented Architecture (SOA) that would require a change of the currently existing data exchange paradigm from pushing data to the European level (EEA) to a ‘data pulling’ setting where data are harvested from a distributed set of national services
- Migration of the approach outside EU, possibly starting from neighbouring countries.

From our perspective, applying the principles of INSPIRE for environmental noise would result in common noise methodologies by reinforcing the accessibility of harmonised and publicly shared data sets. Including those data sets in common noise assessment methodologies and strategic noise mapping practices would increase the harmonisation level of noise assessment and the quality of output maps. INSPIRE standards and principles regarding the interoperability, applied in reporting process would resolve inconsistency and non-comparability issues including cross-border spatial data during data use, re-use and reporting. Strategic noise maps and related action plans, could be assessed on European level with much less efforts compared to the actual situation in which interoperability principles are not applied.

The European SDI and related national nodes can provide dissemination points offering “Information to the public” as required by the END and other environmental policies. The noise maps and related action plans should be easily retrievable through the European geoportals and quickly accessible as interactive web maps, for consultation and revision.

In closing, by adopting INSPIRE based common data-related practices for harmonising and accessing geospatial data, thematic communities are unlocking data so that they can be re-used easily, effectively and in synergy across various thematic domains such as air quality and noise but also transportation, population exposure and human health, marine environment, and biodiversity. Recently, the air quality community has been embracing these practices for INSPIRE-compliant reporting, that provides an efficient way to also integrate noise data with other information to get new insights in support of environmental decision making. Such practice, represents a big potential to decrease administrative burden which is due to numerous reporting requirements, and also to increase efficiency, as reporting information would be stored where can be best managed, maintained and frequently updated while remotely and easily accessed by the actors involved and/or interested at both EU and national level.

## Abbreviations

The following abbreviations are used in this manuscript:

CONOSSOS-EU	Common Noise Assessment Methods in Europe
EEA	European Environment Agency
ETL	Extract-Transform-Load
ENDRM	END Reporting Mechanism
END	Environmental Noise Directive
EC	European Commission
HALE	Humboldt Alignment Editor
ISO	International Standardization Organization
INSPIRE	Infrastructure for Spatial Information in the European Community
JRC	Joint Research Centre, European Commission
MS	Member States of the European Union
OGC	Open Geospatial Consortium
SEIS	Shared Environmental Information System
SDI	Spatial Data Infrastructure
